# Higher social support is associated with lower tinnitus-related disability among institutionalized older adults: a cross-sectional study

**DOI:** 10.3389/fpubh.2026.1886767

**Published:** 2026-09-09

**Authors:** Lan Zhang, Ying-Chun Li, Yan Liu, Man-Man Wang, Hui-Fang Lu

**Affiliations:** 1 School of Medicine, Wuhan University of Science and Technology, Wuhan, Hubei, China; 2 Department of Otorhinolaryngology Head and Neck Surgery, General Hospital of Central Theater Command, Wuhan, Hubei, China; 3 School of Nursing, Hubei University of Medicine, Shiyan, Hubei, China

**Keywords:** chronic subjective tinnitus, long-term care facilities, older adults, perceived social support, tinnitus handicap inventory, tinnitus-related disability

## Abstract

**Background:**

Tinnitus-related disability among older adults in long-term care facilities may reflect not only auditory symptoms but also functional and psychosocial vulnerability. This study examined the association between perceived social support and Tinnitus Handicap Inventory (THI)-defined tinnitus-related disability among older long-term care residents with chronic subjective tinnitus.

**Methods:**

We conducted a cross-sectional study between July and November 2025 among 390 residents aged 60 years or older with chronic subjective tinnitus in long-term care facilities in urban Wuhan, China. Perceived social support was measured using the 13-item Short-form Social Support Appraisals Scale for older adults, and tinnitus-related disability was assessed using the THI. The primary outcome was continuous THI score; severe/catastrophic THI was additionally analyzed as a binary outcome. Ordinal THI severity and moderate-or-worse THI were examined in sensitivity analyses. Multivariable linear, logistic, ordinal logistic, quartile-based, restricted cubic spline, subgroup, and sensitivity analyses were performed with adjustment for demographic, socioeconomic, lifestyle, institutional, clinical, and tinnitus-related covariates.

**Results:**

The mean age was 79.85 years (SD 8.32), and 237 participants (60.8%) were female. Among the covariates included in the fully adjusted model, hypertension was associated with a higher THI score (adjusted *β* = 23.82, 95% CI 19.00 to 28.65; *p* < 0.001). Per 1-SD higher SSA-13 score, the fully adjusted THI total was 5.24 points lower (*β* = −5.24, 95% CI −7.34 to −3.14; *p* < 0.001), and the odds of severe/catastrophic THI were lower (OR = 0.60, 95% CI 0.45 to 0.80; *p* < 0.001). The highest SSA-13 quartile had a lower adjusted THI score than the lowest quartile (*β* = −12.11, 95% CI −17.39 to −6.84), but intermediate-quartile estimates were non-monotonic; the spline nonlinearity test was significant (*p* < 0.001).

**Conclusion:**

Among older adults with chronic subjective tinnitus living in long-term care facilities, higher perceived social support was associated with lower tinnitus-related disability. Because of the cross-sectional design, these findings should be interpreted as evidence of association rather than causation. Brief assessment of perceived social support may help identify residents who need additional psychosocial attention within long-term care settings.

## 1 Introduction

Among older adults, tinnitus can extend beyond an otological complaint and interfere with emotional well-being and everyday functioning (1, 2). A recent systematic review and meta-analysis estimated that approximately 14% of adults experience tinnitus, with prevalence increasing with age and reaching about 24% among older adults (3). However, these population-based estimates do not specifically characterize older adults living in long-term care facilities. Evidence from institutional settings is comparatively limited. One study of institutionalized older adults reported tinnitus in 28.57% of participants (4), but evidence describing tinnitus prevalence and, particularly, standardized tinnitus-related disability in long-term care populations remains limited. Current evidence therefore does not establish whether institutionalized older adults experience tinnitus more frequently or with greater severity than community-dwelling older adults. Long-term care nevertheless represents a distinct and clinically relevant context in which persistent sensory symptoms coexist with multimorbidity, functional vulnerability, altered opportunities for social participation, and reliance on formal and informal sources of support (5–7). Within this context, tinnitus may be relevant not only as a sensory symptom but also in relation to residents’ broader functional, psychosocial, and supportive-care needs.

The impact of tinnitus varies substantially across individuals. Tinnitus has been associated with sleep disturbance, anxiety and depressive symptoms, psychological distress, and poorer health-related quality of life (1, 8–11), but its presence alone does not fully capture its impact on everyday functioning (12, 13). This distinction may be particularly relevant in long-term care settings, where tinnitus is experienced against a background of pre-existing sensory and social vulnerabilities. Hearing impairment is common among residents of long-term care facilities and may add to communication difficulties, while loneliness and variation in perceived social support are well documented in nursing-home populations (7, 14, 15). In this context, tinnitus-related sleep disruption, emotional distress, or difficulty coping with persistent auditory sensations may intersect with existing challenges in communication, social participation, and everyday care. These contextual vulnerabilities should not be interpreted as consequences caused by tinnitus itself; rather, they may shape how tinnitus-related disability is experienced and managed within residential care.

Several standardized instruments have been developed to assess tinnitus-related burden, with different measurement emphases. The Tinnitus Functional Index (TFI) assesses multidimensional functional impact and was designed to be responsive to treatment-related change (16), whereas the Tinnitus Questionnaire (TQ) places greater emphasis on tinnitus-related psychological distress, including cognitive, emotional, and somatic complaints (17). The Tinnitus Handicap Inventory (THI) assesses tinnitus-related handicap across functional, emotional, and catastrophic domains and provides standardized total and severity scores (12, 13, 18). In the present study, the THI was used because a validated Chinese version was available (19) and its focus on tinnitus-related functional and emotional disability aligned with our aim of characterizing the burden of chronic tinnitus among older long-term care residents. For older adults in long-term care settings, THI-defined disability may help identify residents whose tinnitus is associated with functional and emotional care needs, rather than merely documenting symptom presence or loudness (12, 13). Tinnitus commonly coexists with hearing loss in older adults, and comprehensive tinnitus management ideally incorporates audiological evaluation (2, 20, 21). However, this study was designed specifically to characterize self-reported chronic tinnitus and its psychosocial correlates among long-term care residents, and hearing loss was not considered a primary variable of interest at the design stage; objective hearing measures were accordingly not incorporated into the original study protocol.

Perceived social support, a key psychosocial resource for older adults, has been associated with mental health, adaptation to chronic illness, and health-related quality of life (22–24). In long-term care settings, perceived support may arise from several sources, including continued contact with family members, relationships with peer residents, and interactions with formal care staff. These sources are not interchangeable, but together they may shape residents’ perceptions that emotional reassurance, practical assistance, and interpersonal resources are available when needed (15, 25, 26). Perceived social support may be particularly relevant when older residents are managing persistent symptoms such as tinnitus.

The stress-buffering model provides a conceptual rationale for an association between perceived social support and tinnitus-related disability. Rather than implying that support alters the auditory perception itself, the model suggests that the perceived availability of supportive resources may influence how individuals appraise and cope with a persistent stressor (27). In the context of tinnitus, supportive relationships may provide reassurance and opportunities for emotional disclosure, strengthen perceived coping resources and confidence, and help maintain engagement in everyday and social activities (28, 29). These processes may, in turn, influence how much attention is directed toward tinnitus and how intrusive or threatening it is perceived to be (30). This interpretation is also compatible with cognitive-behavioral and biopsychosocial models of tinnitus, which emphasize that tinnitus-related distress and disability are shaped not only by auditory characteristics but also by cognitive appraisal, emotional responses, behavior, and the social environment (28, 30, 31). Importantly, these pathways constitute a conceptual framework for the hypothesized association rather than mediating mechanisms tested in the present study.

This conceptual framework provides a rationale for hypothesizing an inverse association between perceived social support and tinnitus-related disability, although direct empirical evidence remains scarce. Previous studies examining social support in relation to tinnitus outcomes have been conducted mainly in clinical and support-group contexts (28, 29), and some evidence suggests that positive psychological resources such as resilience and self-esteem may be involved in this relationship (29). However, these findings cannot be assumed to generalize directly to older adults living in long-term care facilities, where the organization and availability of social support, caregiving relationships, and everyday care differ from those in community or outpatient settings (15, 26). Whether perceived social support is associated with THI-defined tinnitus-related disability specifically in long-term care therefore remains unclear.

To address this gap, we examined the association between perceived social support and THI-defined tinnitus-related disability among older adults with chronic subjective tinnitus living in long-term care facilities in urban Wuhan, China. We also evaluated whether the association remained robust after additional adjustment for the available self-reported hearing- and tinnitus-care-related variables. We hypothesized that higher perceived social support would be associated with lower tinnitus-related disability.

## 2 Materials and methods

### 2.1 Study design, setting, and participants

This cross-sectional study examined the association between perceived social support and THI-defined tinnitus-related disability among older adults with chronic subjective tinnitus living in long-term care facilities. Reporting followed the STROBE checklist for cross-sectional studies (32).

The study was conducted between July and November 2025 in urban Wuhan, Hubei Province, China. Long-term care facilities were purposively selected from seven central urban administrative districts of Wuhan: Wuchang, Hongshan, Jianghan, Jiang’an, Hanyang, Qingshan, and Qiaokou. Facilities were selected to capture variation in administrative district, ownership, and availability of integrated medical-nursing care, thereby providing institutional heterogeneity across the participating settings. Seventeen eligible facilities were invited; 13 participated, including five public and eight private institutions, with reported resident capacities ranging from 50 to 1,300 residents. No formal *a priori* sample-size calculation was performed. To characterize the statistical sensitivity of the achieved sample for the primary association analysis, a sensitivity power analysis was conducted for the fully adjusted multiple linear regression model. With 390 participants, 356 residual degrees of freedom, a two-sided *α* of 0.05, and 80% power, the minimum detectable incremental effect for perceived social support was Cohen’s *f*^2^ = 0.022, corresponding to a partial *R*^2^ = 0.022.

Within the participating facilities, residents aged 60 years or older were approached and screened for tinnitus symptoms. Residents who reported tinnitus symptoms then underwent further eligibility assessment according to the predefined inclusion and exclusion criteria. Of 3,320 residents contacted, 1,130 reported tinnitus symptoms and proceeded to further eligibility assessment. A total of 420 did not meet the inclusion criteria, 188 met at least one exclusion criterion, and 132 declined to participate. The final analytic sample comprised 390 participants.

The inclusion criteria were: (1) age 60 years or older; (2) residence in a long-term care facility for at least 6 months; (3) chronic subjective tinnitus, defined as self-perceived tinnitus lasting for at least 6 months (33); (4) sufficient cognitive and communicative ability at eligibility screening to understand the survey questions and participate in the interview, either independently or with interviewer assistance; and (5) willingness to participate and provision of written informed consent. This criterion was intended to establish participants’ functional ability to complete the interview rather than to formally classify cognitive status; no standardized cognitive screening instrument was administered.

The exclusion criteria were: (1) documented objective tinnitus or tinnitus attributable to a known identifiable cause; (2) current long-term use of ototoxic or psychotropic medications; and (3) inability, identified during data collection, to complete the interview or provide reliable responses despite interviewer assistance. Chronic subjective tinnitus was identified from participants’ reported symptom history and duration; a standardized otolaryngologic evaluation was not required for study entry.

### 2.2 Measures and covariates

Data were collected using structured face-to-face interviews. When participants had difficulty reading questionnaire items, trained investigators read the items aloud in a neutral manner and recorded the participant’s responses without interpretation or prompting.

A researcher-designed form was used to collect sociodemographic, socioeconomic, lifestyle, institutional, clinical, and tinnitus-related information. Sociodemographic and socioeconomic variables included age, sex, marital status, education level, income, and medical insurance type. Lifestyle variables included smoking history and drinking history, and family visit frequency was also recorded. Institutional variables included facility ownership and the availability of integrated medical-nursing care services. Clinical information included body mass index and hypertension status. Tinnitus-related variables included tinnitus duration, tinnitus pattern, tinnitus location, tinnitus loudness, self-reported hearing loss, hearing aid use, and receipt of professional treatment for tinnitus. Self-reported hearing loss was assessed using a single yes-or-no item. Hearing aid use was recoded as any use versus none for the expanded sensitivity analysis. Objective audiometric assessments and hearing-specific instruments, such as the HHIE/HHIE-S, were not administered. Covariates included in the primary multivariable models were selected *a priori* based on clinical relevance, the long-term care setting, and previous evidence regarding factors associated with tinnitus-related disability (1, 10, 20, 33).

Perceived social support was measured using the 13-item Short-form Social Support Appraisals Scale for older adults (SSA-13) (34, 35). The scale assesses perceived support from family, friends, and others. Each item is scored on a 4-point Likert scale ranging from 1 to 4, and total scores range from 13 to 52, with higher scores indicating greater perceived social support. For regression analyses, the social support score was standardized, and effect estimates were reported per 1-standard-deviation increase in perceived social support.

Tinnitus-related disability was assessed using the THI, developed by Newman et al. (13) and translated into Chinese and validated by Shi (19). The THI contains 25 items, with responses scored as 0 for “no,” 2 for “sometimes,” and 4 for “always.” Total scores range from 0 to 100, with higher scores indicating greater tinnitus-related disability.

The continuous THI total score was used as the primary outcome. THI severity was categorized as slight, 0–16; mild, 18–36; moderate, 38–56; severe, 58–76; and catastrophic, 78–100 (36). In an additional binary analysis, the severe and catastrophic categories were combined to represent the two highest conventional THI severity grades. Because moderate tinnitus-related disability may also be clinically relevant, THI severity was additionally modeled as an ordinal outcome, and moderate-or-worse tinnitus-related disability (THI > 36) was examined as an alternative binary outcome in sensitivity analyses.

### 2.3 Reliability assessment

Internal consistency of the social support scale and the THI was evaluated using Cronbach’s alpha and McDonald’s omega. Reliability coefficients were calculated using the analytic sample.

### 2.4 Statistical analysis

Continuous variables were summarized as mean ± standard deviation or median with interquartile range, as appropriate, and categorical variables were summarized as frequencies and percentages. Participants were grouped according to whether they had severe or catastrophic tinnitus-related disability. Between-group comparisons were performed using independent-samples *t*-tests or Mann–Whitney *U* tests for continuous variables, and chi-square tests or Fisher’s exact tests for categorical variables, as appropriate.

The primary analysis examined the association between perceived social support and continuous THI score using linear regression. A complementary logistic regression analysis examined severe/catastrophic tinnitus-related disability as an additional binary outcome. The social support score was standardized, and regression coefficients and odds ratios were reported with 95% confidence intervals per one-standard-deviation increase in perceived social support. Three sequential models were fitted. Model 1 was unadjusted. Model 2 adjusted for age, sex, and education level. Model 3 further adjusted for body mass index, marital status, income, medical insurance type, smoking history, drinking history, family visit frequency, tinnitus duration, tinnitus pattern, tinnitus location, tinnitus loudness, facility ownership, availability of integrated medical-nursing care services, and hypertension. Heteroscedasticity-consistent HC3 robust standard errors were used for linear regression models. Conventional model-based standard errors were used for the complementary logistic regression models.

To examine whether the association differed across levels of social support, perceived social support was categorized into quartiles, with the lowest quartile used as the reference group. Adjusted mean differences in THI score and adjusted odds ratios for severe/catastrophic tinnitus-related disability were estimated using the fully adjusted model. Restricted cubic spline models were also fitted to assess potential nonlinear associations between perceived social support and both continuous THI score and severe/catastrophic tinnitus-related disability. Nonlinearity was assessed using tests of the nonlinear spline terms.

Exploratory subgroup analyses were conducted to assess whether the association between perceived social support and THI score was generally consistent across clinically relevant strata, including age, sex, family visit frequency, tinnitus duration, tinnitus pattern, facility ownership, availability of integrated medical-nursing care services, hypertension status, and tinnitus loudness. Interaction was assessed by adding multiplicative interaction terms between standardized perceived social support and each subgroup variable. These analyses were considered exploratory and interpreted as hypothesis-generating.

Sensitivity and exploratory analyses were conducted to evaluate the robustness and dimensional consistency of the findings. First, ordinal logistic regression was performed using the five THI severity categories as the outcome. Second, moderate-or-worse tinnitus-related disability, defined as a THI score >36, was used as an alternative binary outcome. Third, in an exploratory analysis, the three validated THI subscales proposed by Newman et al. (13)—functional (11 items), emotional (9 items), and catastrophic (5 items)—were examined as separate continuous outcomes. The same fully adjusted linear regression model used for the THI total score was applied to each subscale, with standardized perceived social support as the primary predictor and HC3 robust standard errors used for inference.

To evaluate the robustness of the association after additional adjustment for available hearing-related factors, the fully adjusted Model 3 was further adjusted for self-reported hearing loss, hearing aid use, and receipt of professional treatment for tinnitus. Because of the small numbers in the original side-specific hearing-aid categories, hearing aid use was recoded as any use versus none. HC3 robust standard errors were applied in the expanded hearing-related models.

Because no missing values were observed for variables included in the analysis, all participants in the analytic sample were included in the complete-case analyses. All statistical tests were two-sided, and *p*-values < 0.05 were considered statistically significant. Analyses were performed using R software version 4.5.3.

## 3 Results

### 3.1 Participant characteristics and scale reliability

A total of 390 older adults with chronic subjective tinnitus were included in the analysis. No missing values were observed for the variables used in this study. The mean age was 79.85 years (SD = 8.32), and 237 participants (60.8%) were female. The mean SSA-13 score was 24.78 (SD = 6.72), and the mean THI score was 55.14 (SD = 21.22).

The SSA-13 showed good internal consistency, with a Cronbach’s alpha of 0.826 and McDonald’s omega of 0.836. The THI showed excellent internal consistency, with a Cronbach’s alpha of 0.910 and McDonald’s omega of 0.917.

According to THI severity categories, 8 participants (2.1%) had slight tinnitus-related disability, 81 (20.8%) had mild disability, 112 (28.7%) had moderate disability, 119 (30.5%) had severe disability, and 70 (17.9%) had catastrophic disability. Overall, 189 participants (48.5%) were classified as having severe or catastrophic tinnitus-related disability.

### 3.2 Characteristics according to severe/catastrophic tinnitus-related disability

Participant characteristics according to severe/catastrophic tinnitus-related disability are shown in Table 1. Participants with severe/catastrophic tinnitus-related disability had lower perceived social support scores than those without severe/catastrophic disability (22.80 ± 5.33 vs. 26.64 ± 7.34, *p* < 0.001). They also had slightly higher tinnitus loudness scores (6.54 ± 1.45 vs. 6.25 ± 1.36, *p* = 0.042), and a higher prevalence of hearing aid use (22.2% vs. 13.4%, *p* = 0.032). Between-group differences were also observed for sex, education level, smoking history, drinking history, tinnitus duration, and hypertension status. Additional participant characteristics and covariates are presented in Supplementary Table S1.

**Table 1:** Characteristics of participants with and without severe/catastrophic tinnitus-related disability.

Characteristic	Overall	Non-severe/catastrophic THI	Severe/catastrophic THI	*p*-value
*N*	390	201	189	
Age, years	79.85 ± 8.32	79.68 ± 8.72	80.03 ± 7.90	0.673
Sex				0.032
Male	153 (39.2%)	68 (33.8%)	85 (45.0%)	
Female	237 (60.8%)	133 (66.2%)	104 (55.0%)	
Education level				<0.001
Primary or below	124 (31.8%)	53 (26.4%)	71 (37.6%)	
Junior middle	102 (26.2%)	48 (23.9%)	54 (28.6%)	
High/vocational	81 (20.8%)	41 (20.4%)	40 (21.2%)	
College	46 (11.8%)	26 (12.9%)	20 (10.6%)	
Bachelor or above	37 (9.5%)	33 (16.4%)	4 (2.1%)	
Smoking history				0.002
No	304 (77.9%)	170 (84.6%)	134 (70.9%)	
Yes	86 (22.1%)	31 (15.4%)	55 (29.1%)	
Drinking history				0.028
No	310 (79.5%)	169 (84.1%)	141 (74.6%)	
Yes	80 (20.5%)	32 (15.9%)	48 (25.4%)	
Family visit frequency				0.660
1–2/week	153 (39.2%)	79 (39.3%)	74 (39.2%)	
1–2/month	141 (36.2%)	68 (33.8%)	73 (38.6%)	
1–2/quarter	41 (10.5%)	23 (11.4%)	18 (9.5%)	
1–2/half-year	35 (9.0%)	18 (9.0%)	17 (9.0%)	
1–2/year	20 (5.1%)	13 (6.5%)	7 (3.7%)	
Tinnitus duration				<0.001
0.5–1 year	77 (19.7%)	29 (14.4%)	48 (25.4%)	
1–5 years	115 (29.5%)	49 (24.4%)	66 (34.9%)	
5–10 years	133 (34.1%)	65 (32.3%)	68 (36.0%)	
≥10 years	65 (16.7%)	58 (28.9%)	7 (3.7%)	
Tinnitus pattern				0.327
Continuous	138 (35.4%)	66 (32.8%)	72 (38.1%)	
Intermittent	252 (64.6%)	135 (67.2%)	117 (61.9%)	
Tinnitus loudness	6.39 ± 1.41	6.25 ± 1.36	6.54 ± 1.45	0.042
Self-reported hearing loss				0.837
No	104 (26.7%)	55 (27.4%)	49 (25.9%)	
Yes	286 (73.3%)	146 (72.6%)	140 (74.1%)	
Hearing aid use				0.032
No	321 (82.3%)	174 (86.6%)	147 (77.8%)	
Yes	69 (17.7%)	27 (13.4%)	42 (22.2%)	
Receipt of professional treatment for tinnitus				1.000
No	273 (70.0%)	141 (70.1%)	132 (69.8%)	
Yes	117 (30.0%)	60 (29.9%)	57 (30.2%)	
Institutional ownership				0.521
Public	234 (60.0%)	117 (58.2%)	117 (61.9%)	
Private	156 (40.0%)	84 (41.8%)	72 (38.1%)	
Integrated medical-nursing care status				0.787
Yes	184 (47.2%)	93 (46.3%)	91 (48.1%)	
No	206 (52.8%)	108 (53.7%)	98 (51.9%)	
Hypertension				<0.001
No	288 (73.8%)	184 (91.5%)	104 (55.0%)	
Yes	102 (26.2%)	17 (8.5%)	85 (45.0%)	
Social support score	24.78 ± 6.72	26.64 ± 7.34	22.80 ± 5.33	<0.001
THI score	55.14 ± 21.22	37.15 ± 11.03	74.26 ± 9.42	<0.001

THI, Tinnitus Handicap Inventory; SD, standard deviation. Data are presented as mean ± SD or *n* (%), as appropriate. *P*-values were calculated to compare participants with and without severe/catastrophic tinnitus-related disability. The three hearing-related variables were included in the expanded sensitivity analysis but not in the original primary Model 3.

### 3.3 Association between social support and tinnitus-related disability

In linear regression analyses, perceived social support was inversely associated with THI scores. In the unadjusted model, each 1-SD increase in perceived social support corresponded to an 8.44-point lower THI score (95% CI: −10.40 to −6.49, *p* < 0.001). This association was slightly attenuated after adjustment for age, sex, and education level (*β* = −8.05, 95% CI: −10.19 to −5.92, *p* < 0.001) and remained significant in the fully adjusted model (*β* = −5.24, 95% CI: −7.34 to −3.14, *p* < 0.001). Similar findings were observed in logistic regression analyses, where each 1-SD increase in perceived social support was associated with lower odds of severe/catastrophic tinnitus-related disability in the fully adjusted model (OR = 0.60, 95% CI: 0.45 to 0.80, *p* < 0.001; Table 2). In addition, among the covariates included in the fully adjusted linear regression model, hypertension was associated with a higher THI score (adjusted *β* = 23.82, 95% CI 19.00 to 28.65; *p* < 0.001).

**Table 2:** Main regression models for the association between social support and tinnitus-related disability.

Outcome	Model	Effect estimate	95% CI	*p*-value
THI score	Unadjusted	*β* = −8.44	−10.40 to −6.49	<0.001
	Demographic/socioeconomic adjusted	*β* = −8.05	−10.19 to −5.92	<0.001
	Fully adjusted	*β* = −5.24	−7.34 to −3.14	<0.001
Severe/catastrophic tinnitus-related disability	Unadjusted	OR = 0.54	0.43 to 0.67	<0.001
	Demographic/socioeconomic adjusted	OR = 0.55	0.43 to 0.70	<0.001
	Fully adjusted	OR = 0.60	0.45 to 0.80	<0.001

THI, Tinnitus Handicap Inventory; CI, confidence interval; OR, odds ratio. *β* values represent differences in THI total score per 1-SD higher perceived social support score, and ORs represent the odds of severe/catastrophic THI per 1-SD higher perceived social support score. Model 1 was unadjusted. Model 2 adjusted for age, sex, and education level. Model 3 further adjusted for body mass index, marital status, income, medical insurance type, smoking history, drinking history, family visit frequency, tinnitus duration, tinnitus pattern, tinnitus location, tinnitus loudness, facility ownership, availability of integrated medical-nursing care services, and hypertension. Confidence intervals for the primary linear regression models were based on HC3 robust standard errors; those for the logistic regression models were based on conventional model-based standard errors.

To further characterize this association across different dimensions of tinnitus-related disability, the functional, emotional, and catastrophic THI subscales were examined separately. In the fully adjusted models, each 1-SD increase in perceived social support was associated with lower functional (*β* = −2.15, 95% CI: −3.18 to −1.12, *p* < 0.001), emotional (*β* = −1.98, 95% CI: −2.87 to −1.10, *p* < 0.001), and catastrophic subscale scores (*β* = −1.10, 95% CI: −1.56 to −0.64, *p* < 0.001). The standardized coefficients were similar in magnitude across the three subscales (standardized *β* = −0.216, −0.234, and −0.230, for the functional, emotional, and catastrophic subscales, respectively; Supplementary Table S3).

### 3.4 Categorical and non-linear analyses of social support

In quartile-based analyses, the association between perceived social support and tinnitus-related disability was not monotonic. The prevalence of severe/catastrophic tinnitus-related disability was 66 (55.0%) in Q1, 55 (65.5%) in Q2, 54 (54.0%) in Q3, and 14 (16.3%) in Q4. Compared with Q1, Q4 was associated with a lower adjusted THI score (*β* = −12.11, 95% CI: −17.39 to −6.84, *p* < 0.001) and lower odds of severe/catastrophic tinnitus-related disability (OR = 0.25, 95% CI: 0.11 to 0.61, *p* = 0.002). Estimates for Q2 and Q3 did not indicate a graded reduction in tinnitus-related disability (Table 3).

**Table 3:** Association between social support quartiles and tinnitus-related disability.

Social support quartile	Score range	*n*	THI score, mean ± SD	Severe/catastrophic THI, n (%)	Adjusted β (95% CI)	*p*-value	Adjusted OR (95% CI)	*p*-value
Q1	13–20	120	62.47 ± 22.84	66 (55.0%)	Reference	Reference	Reference	Reference
Q2	21–24	84	60.74 ± 20.26	55 (65.5%)	2.78 (−2.69 to 8.24)	0.320	2.96 (1.29 to 6.83)	0.011
Q3	25–30	100	53.96 ± 16.70	54 (54.0%)	−0.81 (−6.11 to 4.50)	0.766	2.04 (0.94 to 4.42)	0.071
Q4	31–44	86	40.81 ± 17.00	14 (16.3%)	−12.11 (−17.39 to −6.84)	<0.001	0.25 (0.11 to 0.61)	0.002

THI, Tinnitus Handicap Inventory; SD, standard deviation; CI, confidence interval; OR, odds ratio; SSA-13, 13-item Short-form Social Support Appraisals Scale. Quartiles were based on the observed distribution of SSA-13 scores in the analytic sample. Q1 was the reference group. β values indicate adjusted differences in THI score, and ORs indicate adjusted odds of severe/catastrophic tinnitus-related disability, both compared with Q1. Models were adjusted for demographic, socioeconomic, lifestyle, institutional, clinical, and tinnitus-related covariates. Quartile-based estimates were not monotonic and should not be interpreted as a graded dose–response association.

**Figure 1:**
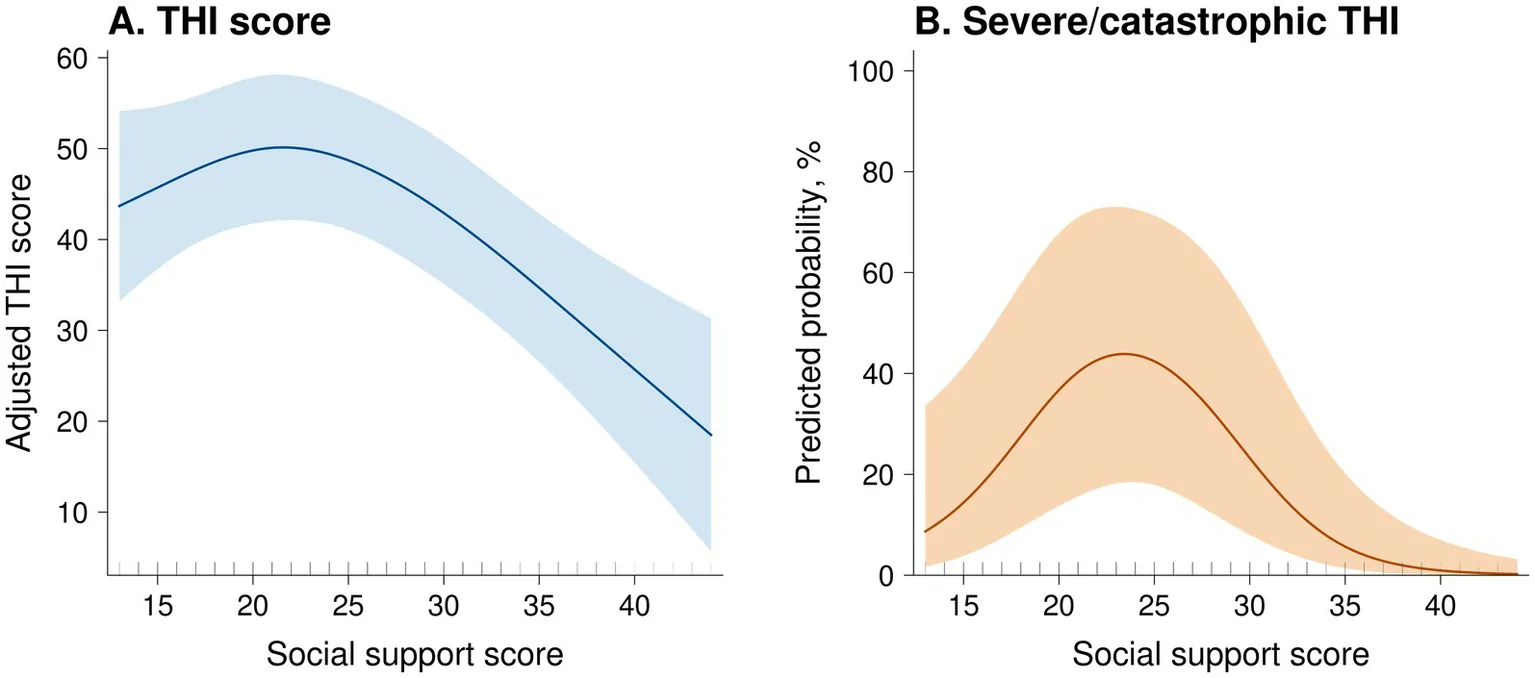
Restricted cubic spline analyses of social support and tinnitus-related disability. Panel **(A)** shows the fully adjusted association between social support score and THI score. Panel **(B)** shows the fully adjusted association between social support score and severe/catastrophic THI. Shaded areas indicate 95% confidence bands. Models were adjusted for demographic, socioeconomic, lifestyle, institutional, clinical, and tinnitus-related covariates. *P* for non-linearity was <0.001 for panel **(A)** and <0.001 for panel **(B)**.

Restricted cubic spline models provided evidence of nonlinearity for both continuous THI score and severe/catastrophic tinnitus-related disability (both *p* for non-linearity < 0.001; Figure 1), suggesting that the association was unlikely to follow a simple linear dose–response pattern.

### 3.5 Subgroup and sensitivity analyses

**Figure 2:**
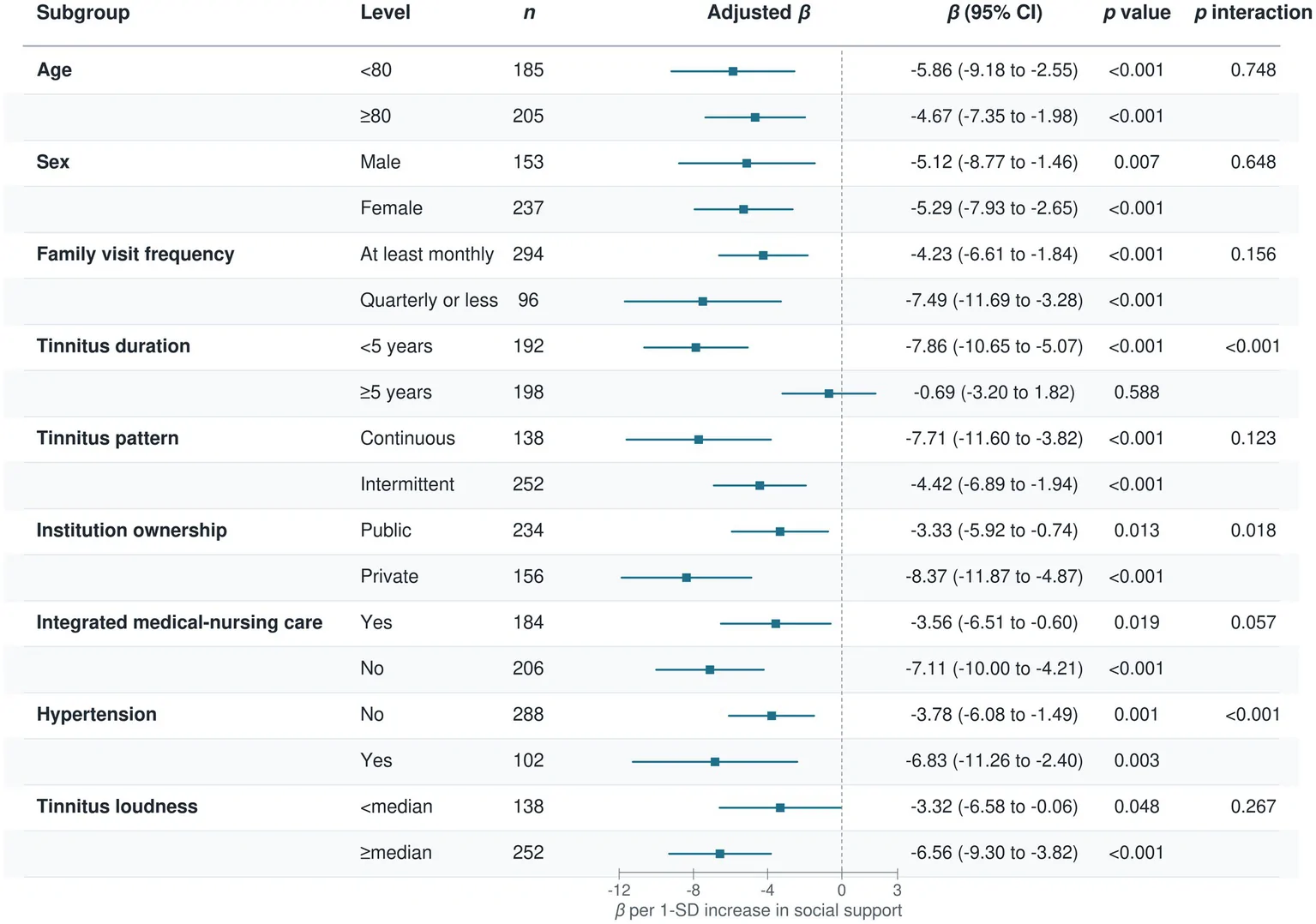
Exploratory subgroup analyses for the association between perceived social support and THI score. Points indicate adjusted *β* values per 1-standard-deviation higher perceived social support score, and horizontal lines indicate 95% CIs. *P* for interaction values were obtained from multiplicative interaction terms. No adjustment was made for multiple comparisons.

Subgroup analyses are shown in Figure 2. The inverse association between perceived social support and THI score was observed in most strata. Interaction tests suggested possible variation by tinnitus duration, institutional ownership, and hypertension status. Given the number of subgroup comparisons and the limited sample size in some strata, these findings were considered exploratory.

Sensitivity analyses using alternative THI outcome definitions yielded results consistent with the primary continuous-outcome analysis. In the ordinal logistic regression model, higher perceived social support was associated with lower odds of being in a more severe THI category (OR = 0.56, 95% CI: 0.45 to 0.70, *p* < 0.001). Similar results were observed when moderate-or-worse tinnitus-related disability was analyzed as an alternative binary outcome (OR = 0.59, 95% CI: 0.43–0.80, *p* < 0.001; Table 4).

**Table 4:** Sensitivity analyses.

Analysis	Outcome definition	Effect estimate	95% CI	*p*-value
Ordinal logistic regression	THI severity categories	OR = 0.56	0.45 to 0.70	<0.001
Alternative binary outcome logistic regression	Moderate-or-worse THI (>36)	OR = 0.59	0.43 to 0.80	<0.001

THI, Tinnitus Handicap Inventory; CI, confidence interval; OR, odds ratio. All models used the fully adjusted covariate set. Ordinal logistic regression modeled the five THI severity categories. The alternative binary outcome was defined as moderate-or-worse tinnitus-related disability (THI >36).

### 3.6 Robustness to hearing-related covariates

In an expanded sensitivity analysis, the fully adjusted models were additionally adjusted for self-reported hearing loss, hearing aid use, and receipt of professional treatment for tinnitus, with HC3 robust standard errors applied. The association between perceived social support and THI-defined disability remained materially unchanged: per 1-SD higher SSA-13 score, *β* = −5.39 (95% CI: −7.49 to −3.28, *p* < 0.001) for THI total score, OR = 0.59 (95% CI: 0.40 to 0.87, *p* = 0.007) for moderate-or-worse THI, and OR = 0.58 (95% CI: 0.42 to 0.80, *p* < 0.001) for severe/catastrophic THI. None of the three hearing-related variables was individually associated with any outcome (all *p* > 0.05). Joint HC3-robust Wald tests of the three hearing-related variables were non-significant for THI total score, moderate-or-worse THI, and severe/catastrophic THI (*p* = 0.208, 0.301, and 0.159, respectively; Supplementary Table S2).

## 4 Discussion

### 4.1 Principal findings

In this cross-sectional study of older adults with chronic subjective tinnitus living in long-term care facilities, higher perceived social support was associated with less THI-defined tinnitus-related disability. The continuous THI total score was the primary outcome, while severe/catastrophic THI was additionally examined as a binary outcome representing the two highest conventional THI severity grades (36). Moderate tinnitus-related disability is also clinically relevant; therefore, moderate-or-worse THI and the full ordinal THI severity classification were examined in sensitivity analyses. The association was consistent across these alternative outcome definitions, suggesting that the findings were not dependent on exclusion of the moderate category from the severe/catastrophic binary definition.

In the fully adjusted linear regression model, a 1-standard-deviation higher perceived social support score corresponded to a 5.24-point lower THI score. This difference was slightly smaller than the previously reported minimal clinically relevant change of approximately 6–7 points for the THI (37). Because that threshold was developed for within-person change, whereas our estimate reflects a cross-sectional between-person association, the clinical meaning for individual residents should be interpreted cautiously. At the group level, however, the consistency across outcome definitions suggests that perceived social support may help distinguish residents with a lower burden of tinnitus-related disability.

### 4.2 Interpretation in relation to previous research

Tinnitus-related burden is not determined by auditory characteristics alone. Current tinnitus guidance and biopsychosocial models emphasize that tinnitus-related distress and disability reflect interactions among auditory and clinical characteristics, psychological responses, and the social environment (20, 21, 30, 31, 33, 38). The present study was not designed as a formal test of the biopsychosocial model, but several measured variables can be situated within this broader framework. Tinnitus duration, loudness, pattern, location, self-reported hearing loss, and comorbidity represented aspects of the auditory and clinical context, whereas perceived social support, family visit frequency, facility ownership, and integrated medical–nursing care reflected elements of the social and care context. Psychological processes such as anxiety, depression, coping, loneliness, and perceived control were not directly measured and therefore remain plausible but untested pathways.

This interpretation is consistent with previous tinnitus-specific research. Wang et al. (29) reported that resilience and self-esteem mediated the association between social support and tinnitus distress in patients with chronic tinnitus, suggesting that psychological resources may be relevant to how supportive relationships are translated into lower symptom-related distress. Within long-term care, perceived support may arise through several contextually distinct sources. Continued family support may provide emotional continuity, familiarity, reassurance, and opportunities for residents to disclose tinnitus-related concerns (25). Relationships with peer residents may provide companionship and participation in shared activities, potentially reducing social isolation and sustained attention to persistent tinnitus sensations. Formal support from nurses and other care staff may contribute through acknowledgement of symptoms, practical assistance, reassurance, and facilitation of access to hearing or tinnitus-related care when symptoms interfere with everyday functioning. Together, these supportive experiences may influence how residents appraise, cope with, and accommodate persistent tinnitus even when the auditory percept itself remains unchanged (27–29). These pathways are consistent with the stress-buffering perspective and with cognitive-behavioral and biopsychosocial models of tinnitus, which emphasize the roles of appraisal, emotional responses, behavior, attention, and the social environment in tinnitus-related disability (27, 30, 39).

These source-specific pathways were not measured separately in the present study. The SSA-13 captures residents’ overall perceptions of available support from family, friends, and others rather than distinct effects attributable to family members, peer residents, or care staff. The similar inverse associations observed across the functional, emotional, and catastrophic THI domains nevertheless suggest that the relationship with perceived support was not confined to emotional distress alone. Within a biopsychosocial interpretation, social resources may also be relevant to everyday functioning, symptom appraisal, and perceived coping resources. These findings should not, however, be interpreted as evidence that any particular source of support causally reduces tinnitus-related disability.

Because the present cross-sectional data do not establish temporal ordering and did not include direct measures of the proposed psychosocial pathways, mediation analysis was not performed. Any mediation model fitted to these data would risk implying mechanistic or temporal relationships that the study design cannot establish. Future longitudinal studies should evaluate these pathways using temporally ordered assessments of loneliness, coping, psychological resilience, perceived control, emotional distress, sleep disturbance, and social participation, together with objective hearing assessment and relevant facility-level care characteristics.

### 4.3 Exploratory analysis of non-linear associations

The quartile-based and restricted cubic spline analyses did not support a simple graded dose–response relationship between perceived social support and tinnitus-related disability. The clearest separation was observed in the highest quartile, in which residents had substantially lower THI scores and a lower prevalence of severe/catastrophic disability, whereas estimates for the two intermediate quartiles were irregular. In particular, the second quartile did not show an intermediate reduction in tinnitus-related disability and was associated with higher odds of severe/catastrophic disability relative to the lowest quartile. Together with the significant spline tests for nonlinearity, this pattern suggests that the association may vary across the distribution of perceived support rather than increasing uniformly with each incremental level of support.

One possible interpretation in the context of long-term care is that the relevance of perceived support may depend not simply on the amount of support available, but on whether support is sufficiently consistent, dependable, and accessible in everyday life. Residents reporting relatively high overall support may be more likely to experience sustained reassurance, opportunities for symptom disclosure, assistance with daily difficulties, and continued engagement with family, peers, or staff. By contrast, intermediate total scores may encompass heterogeneous support situations—for example, relatively strong support from one source but limited or inconsistent support from others—which may not translate into the same perceived security or continuity of support. The SSA-13 total score cannot distinguish these specific patterns, however, so this explanation remains hypothesis-generating rather than demonstrated by the present data.

Importantly, the boundary of the highest quartile should not be interpreted as a clinical threshold or a target score for intervention. The quartile cut points were derived from the score distribution in this sample, and the irregular estimates in Q2 and Q3 may also reflect residual confounding, sample-specific score distributions, or chance. The nonlinear findings therefore support caution against assuming a uniform dose–response relationship and instead suggest that future studies should examine whether the consistency, source, and accessibility of support help explain why very high perceived support was associated with distinctly lower tinnitus-related disability in this sample.

### 4.4 Implications for long-term care practice

Exploratory subgroup analyses showed that the inverse association between perceived social support and THI score was generally present across most participant groups. The association appeared stronger among residents with shorter tinnitus duration, those living in private facilities, those in facilities without integrated medical-nursing care services, and those with hypertension. These patterns may reflect differences in symptom course, care environment, access to integrated services, or comorbidity burden, but they should not be interpreted as firm evidence of effect modification. Because sample sizes within individual strata were limited and multiple comparisons were performed, the subgroup findings are best viewed as exploratory and should be examined in future studies.

From an aging and long-term care perspective, these findings suggest that tinnitus-related disability should not be evaluated only by tinnitus duration, loudness, or the presence of tinnitus symptoms. THI scores capture the functional and emotional burden of tinnitus and may provide information that is more relevant to daily care planning for older residents (12, 13). Adding a brief assessment of perceived social support may help identify residents who are more vulnerable to tinnitus-related disability and who may require additional psychosocial attention.

The findings also highlight the role of the long-term care environment. In institutional settings, perceived support is shaped not only by individual relationships but also by family contact, peer interaction, staff responsiveness, and daily care routines. Support-oriented strategies may therefore be considered in routine care, including regular communication with family members, opportunities for peer interaction, staff awareness of tinnitus-related distress, and referral pathways for residents with substantial THI burden. These approaches should complement, rather than replace, audiological assessment and clinical tinnitus management. Consistent with international tinnitus practice guidelines, residents reporting substantial tinnitus-related disability may warrant otolaryngology or audiology review; assessment for treatable otologic causes; audiological evaluation, including pure-tone and speech testing; assessment of hearing-aid and hearing-rehabilitation needs; tinnitus education and counseling; and screening for co-occurring sleep, anxiety, or depressive symptoms, with graded referral when clinically indicated (20, 21, 33, 38). Future intervention studies should evaluate whether these graded, multidisciplinary rehabilitation strategies improve tinnitus-related disability and related functional outcomes among institutionalized older adults.

### 4.5 Strengths and limitations

This study has several strengths. It focused on institutionalized older adults with chronic subjective tinnitus, a population that remains underrepresented in tinnitus research. Tinnitus-related disability was assessed using the THI rather than relying only on tinnitus presence, duration, or loudness. In addition, the association between perceived social support and tinnitus-related disability was evaluated across multiple outcome definitions, including continuous THI score, severe/catastrophic THI as an additional binary outcome, and ordinal THI severity and moderate-or-worse THI as sensitivity outcomes. The use of quartile-based, nonlinear, subgroup, and sensitivity analyses also allowed the stability and shape of the association to be examined more fully.

Several limitations should be acknowledged. First, the cross-sectional design precludes causal and temporal inference. The direction of the observed association cannot be determined, and reverse causation is possible because greater tinnitus burden may reduce social participation and perceived support (39, 40). Second, the participating long-term care facilities were purposively recruited from seven central urban districts of Wuhan. Although this approach allowed inclusion of public and private facilities with differing care arrangements, it did not constitute probability-based sampling of all long-term care facilities in Wuhan. Generalization to suburban, county-level, rural, or other long-term care settings should therefore be made cautiously. In addition, tinnitus ascertainment relied on participant-reported symptoms, and eligibility required sufficient cognitive and communicative ability to participate in the interview. These features may have influenced the composition of the analytic sample and should be considered when extrapolating the findings beyond the enrolled population. Third, tinnitus-related disability and perceived social support were assessed using self-report instruments, and responses may have been influenced by recall bias, response style, or interviewer-assisted completion. Fourth, chronic subjective tinnitus was identified from participant-reported symptoms, consistent with its inherently subjective nature; however, standardized otolaryngologic evaluation was not performed, the available hearing-related variables were based on self-report, and objective audiometric data were unavailable. Nevertheless, additional adjustment for self-reported hearing loss, hearing aid use, and receipt of professional tinnitus treatment did not materially alter the association between perceived social support and THI-defined disability. In addition, although individual receipt of professional tinnitus treatment was recorded, the availability and use of hearing- and tinnitus-rehabilitation services at the facility level were not systematically assessed. Future studies should incorporate otolaryngologic evaluation, pure-tone audiometry, and, where appropriate, hearing-specific questionnaires such as the HHIE-S, together with more detailed assessment of rehabilitation-service availability and use. Fifth, cognitive function was not assessed using a standardized screening instrument. Eligibility was based on participants’ functional ability to understand the survey questions and communicate responses during the interview; therefore, some inter-rater variability in eligibility assessment cannot be excluded, and residents with cognitive or communication difficulties may have been underrepresented. Standardized measures of depressive symptoms, anxiety, sleep, and facility-related quality of life were also unavailable, leaving the possibility of residual confounding by these and other unmeasured psychosocial or clinical factors. Sixth, although the severe/catastrophic grouping represented the two highest conventional THI severity grades, dichotomizing THI necessarily simplified the underlying severity continuum. Moderate tinnitus-related disability may also be clinically relevant, and the severe/catastrophic grouping should therefore not be interpreted as a universally validated clinical referral or treatment threshold. Nevertheless, the consistency of the findings across continuous THI score, ordinal severity categories, and the alternative moderate-or-worse threshold suggests that the observed association was not dependent on a single cutoff definition. Finally, the nonlinear and subgroup analyses were exploratory and should be regarded as hypothesis-generating.

## 5 Conclusion

In this cross-sectional study of older adults with chronic subjective tinnitus living in long-term care facilities, higher perceived social support was consistently associated with lower tinnitus-related disability across continuous, binary, and ordinal THI outcome definitions. The findings suggest that social support may be an important psychosocial correlate of tinnitus-related disability in long-term care settings. However, the cross-sectional design precludes causal inference, and the absence of objective audiometric and standardized psychological measures leaves potential residual confounding. Future longitudinal studies incorporating mental health assessment, hearing evaluation, and facility-level care characteristics are needed to clarify whether perceived social support predicts subsequent changes in tinnitus-related disability in this population.

## Data Availability

The raw data supporting the conclusions of this article will be made available by the authors, without undue reservation, upon reasonable request to the corresponding author.
