# Supplementary material for: Higher social support is associated with lower tinnitus-related disability among institutionalized older adults: a cross-sectional study
Source: Front Public Health. 2026 Sep 9;14:1886767. doi: 10.3389/fpubh.2026.1886767 (PMC13597730; doi:10.3389/fpubh.2026.1886767)
Supplement: Supplementary file 1 [file Supplementary_file_1.docx]

**FIG1 final complete R code**

Standalone code for the final two-panel restricted cubic spline figure. Generated: 2026-05-10 22:56

```
# Final FIG1 code: restricted cubic spline analyses and two-panel Figure 1 export.
# Source basis: diagnose_table3_reanalysis.R, final RCS section.

options(stringsAsFactors = FALSE)

candidate_libs <- c(
  "D:/tinnitus/R_local_lib",
  file.path(getwd(), "R_lib")
)
candidate_libs <- candidate_libs[dir.exists(candidate_libs)]
if (length(candidate_libs) > 0) {
  .libPaths(c(candidate_libs, .libPaths()))
}

suppressPackageStartupMessages({
  library(haven)
  library(dplyr)
  library(tidyr)
  library(readr)
  library(stringr)
  library(ggplot2)
  library(sandwich)
  library(lmtest)
  library(broom)
  library(car)
  library(MASS)
  library(splines)
  library(patchwork)
})

select <- dplyr::select
rename <- dplyr::rename
filter <- dplyr::filter
mutate <- dplyr::mutate

out_dir <- file.path(getwd(), "table3_diagnostic_results")
dir.create(out_dir, showWarnings = FALSE, recursive = TRUE)

fmt_p <- function(p) {
  ifelse(is.na(p), NA_character_, ifelse(p < 0.001, "<0.001", sprintf("%.3f", p)))
}

fmt_num <- function(x, digits = 2) {
  ifelse(is.na(x), NA_character_, sprintf(paste0("%.", digits, "f"), x))
}

ci_text <- function(lo, hi, digits = 2) {
  paste0(fmt_num(lo, digits), " to ", fmt_num(hi, digits))
}

mode_value <- function(x) {
  ux <- na.omit(x)
  if (length(ux) == 0) return(NA)
  tab <- sort(table(ux), decreasing = TRUE)
  names(tab)[1]
}

make_factor <- function(x, labels) {
  factor(as.integer(x), levels = seq_along(labels), labels = labels)
}

safe_chisq_p <- function(tab) {
  out <- suppressWarnings(chisq.test(tab))
  if (any(out$expected < 5)) {
    suppressWarnings(fisher.test(tab, simulate.p.value = TRUE, B = 10000)$p.value)
  } else {
    out$p.value
  }
}

cramers_v <- function(tab) {
  suppressWarnings({
    chi <- chisq.test(tab, correct = FALSE)$statistic
    n <- sum(tab)
    denom <- n * (min(dim(tab)) - 1)
    if (denom <= 0) return(NA_real_)
    as.numeric(sqrt(chi / denom))
  })
}

eta_sq <- function(y, group) {
  fit <- lm(y ~ group)
  a <- anova(fit)
  as.numeric(a$`Sum Sq`[1] / sum(a$`Sum Sq`))
}

write_csv_out <- function(x, name) {
  readr::write_csv(x, file.path(out_dir, name), na = "")
}

tidy_lm_terms <- function(model, terms, model_label) {
  sm <- summary(model)$coef
  n_model <- nobs(model)
  purrr::map_dfr(terms, function(term) {
    if (!term %in% rownames(sm)) {
      return(tibble(
        model = model_label, term = term, beta = NA_real_, ci_low = NA_real_,
        ci_high = NA_real_, p = NA_real_, n = n_model
      ))
    }
    est <- sm[term, "Estimate"]
    se <- sm[term, "Std. Error"]
    tibble(
      model = model_label,
      term = term,
      beta = est,
      ci_low = est - 1.96 * se,
      ci_high = est + 1.96 * se,
      p = sm[term, "Pr(>|t|)"],
      n = n_model
    )
  })
}

tidy_glm_terms <- function(model, terms, model_label) {
  sm <- summary(model)$coef
  n_model <- nobs(model)
  purrr::map_dfr(terms, function(term) {
    if (!term %in% rownames(sm)) {
      return(tibble(
        model = model_label, term = term, log_or = NA_real_, OR = NA_real_,
        ci_low = NA_real_, ci_high = NA_real_, p = NA_real_, n = n_model
      ))
    }
    est <- sm[term, "Estimate"]
    se <- sm[term, "Std. Error"]
    tibble(
      model = model_label,
      term = term,
      log_or = est,
      OR = exp(est),
      ci_low = exp(est - 1.96 * se),
      ci_high = exp(est + 1.96 * se),
      p = sm[term, "Pr(>|z|)"],
      n = n_model
    )
  })
}

fit_lm_q <- function(covars, data) {
  rhs <- paste(c("social_support_q", covars), collapse = " + ")
  lm(as.formula(paste("thi ~", rhs)), data = data)
}

fit_glm_q <- function(covars, data) {
  rhs <- paste(c("social_support_q", covars), collapse = " + ")
  glm(as.formula(paste("high_thi ~", rhs)), data = data, family = binomial())
}

fit_trend <- function(outcome, trend_var, covars, family = NULL, data) {
  rhs <- paste(c(trend_var, covars), collapse = " + ")
  f <- as.formula(paste(outcome, "~", rhs))
  if (is.null(family)) lm(f, data = data) else glm(f, data = data, family = family)
}

sav_path <- list.files(".", pattern = "\\.sav$", recursive = TRUE, full.names = TRUE)[1]
if (is.na(sav_path)) stop("No .sav source file found.")
raw <- as.data.frame(read_sav(sav_path))

dat <- data.frame(
  id = as.integer(raw[[1]]),
  age = as.numeric(raw[[2]]),
  age_group = make_factor(raw[[3]], c("60-69", "70-79", "80-89", ">=90")),
  sex = make_factor(raw[[4]], c("Male", "Female")),
  bmi = as.numeric(raw[[5]]),
  marital = make_factor(raw[[7]], c("Unmarried", "Married", "Divorced", "Widowed")),
  education = make_factor(raw[[8]], c("Primary or below", "Junior middle", "High/vocational", "College", "Bachelor or above")),
  family_visit = make_factor(raw[[9]], c("1-2/week", "1-2/month", "1-2/quarter", "1-2/half-year", "1-2/year")),
  income = make_factor(raw[[11]], c("<2000", "2000-4000", "4000-6000", ">6000")),
  insurance = make_factor(raw[[12]], c("Self-pay", "Resident insurance", "Employee insurance")),
  occupation = make_factor(raw[[13]], c("Worker", "Farmer", "Teacher", "Accountant", "Administrative/management", "Other")),
  institution = make_factor(raw[[14]], c("Public", "Private")),
  integrated_care = make_factor(raw[[15]], c("Yes", "No")),
  smoking = make_factor(raw[[16]], c("No", "Yes")),
  drinking = make_factor(raw[[19]], c("No", "Yes")),
  tinnitus_loudness = as.numeric(raw[[22]]),
  tinnitus_location = make_factor(raw[[25]], c("Left ear only", "Right ear only", "Bilateral", "Cranial tinnitus")),
  tinnitus_pattern = make_factor(raw[[26]], c("Continuous", "Intermittent")),
  tinnitus_duration = make_factor(raw[[27]], c("0.5-1 year", "1-5 years", "5-10 years", ">=10 years")),
  hypertension = factor(as.integer(raw[[37]]), levels = c(0, 1), labels = c("No", "Yes")),
  social_support = as.numeric(raw[[122]]),
  thi = as.numeric(raw[[124]])
)

dat$z_age <- as.numeric(scale(dat$age))
dat$z_bmi <- as.numeric(scale(dat$bmi))
dat$z_loudness <- as.numeric(scale(dat$tinnitus_loudness))

dat$thi_severity <- cut(
  dat$thi,
  breaks = c(-Inf, 16, 36, 56, 76, Inf),
  labels = c("Slight", "Mild", "Moderate", "Severe", "Catastrophic"),
  right = TRUE,
  ordered_result = TRUE
)
dat$high_thi <- as.integer(dat$thi > 56)
dat$high_thi_from_severity <- as.integer(dat$thi_severity %in% c("Severe", "Catastrophic"))

q_breaks <- unique(as.numeric(quantile(dat$social_support, probs = seq(0, 1, 0.25), na.rm = TRUE, type = 7)))
dat$social_support_q <- cut(
  dat$social_support,
  breaks = q_breaks,
  include.lowest = TRUE,
  ordered_result = FALSE,
  labels = paste0("Q", seq_len(length(q_breaks) - 1))
)
dat$social_support_q <- factor(dat$social_support_q, levels = paste0("Q", 1:4))
dat$social_support_q_num <- as.integer(dat$social_support_q)
quartile_medians <- tapply(dat$social_support, dat$social_support_q, median)
dat$social_support_q_median <- as.numeric(quartile_medians[as.character(dat$social_support_q)])

dat$tertile <- cut(
  dat$social_support,
  breaks = unique(as.numeric(quantile(dat$social_support, probs = c(0, 1 / 3, 2 / 3, 1), na.rm = TRUE))),
  include.lowest = TRUE,
  labels = paste0("T", 1:3)
)
dat$tertile <- factor(dat$tertile, levels = paste0("T", 1:3))
dat$median_split <- factor(
  ifelse(dat$social_support <= median(dat$social_support), "Low (<=median)", "High (>median)"),
  levels = c("Low (<=median)", "High (>median)")
)


continuous_covars <- c("age", "bmi", "tinnitus_loudness")
categorical_covars <- c(
  "sex", "marital", "education", "income", "insurance", "smoking", "drinking",
  "family_visit", "tinnitus_duration", "tinnitus_pattern", "tinnitus_location",
  "institution", "integrated_care", "hypertension"
)


model_terms <- list(
  "Model 0: unadjusted" = character(0),
  "Model 1: age + sex" = c("z_age", "sex"),
  "Model 2: Model 1 + education" = c("z_age", "sex", "education"),
  "Model 3: Model 2 + smoking + drinking" = c("z_age", "sex", "education", "smoking", "drinking"),
  "Model 4: Model 3 + family visit frequency" = c("z_age", "sex", "education", "smoking", "drinking", "family_visit"),
  "Model 5: Model 4 + tinnitus duration + pattern + loudness" = c("z_age", "sex", "education", "smoking", "drinking", "family_visit", "tinnitus_duration", "tinnitus_pattern", "z_loudness"),
  "Model 6: Model 5 + institution + integrated care" = c("z_age", "sex", "education", "smoking", "drinking", "family_visit", "tinnitus_duration", "tinnitus_pattern", "z_loudness", "institution", "integrated_care"),
  "Model 7: Model 6 + hypertension" = c("z_age", "sex", "education", "smoking", "drinking", "family_visit", "tinnitus_duration", "tinnitus_pattern", "z_loudness", "institution", "integrated_care", "hypertension"),
  "Model 8: exact manuscript fully adjusted" = c("z_age", "sex", "z_bmi", "marital", "education", "income", "insurance", "family_visit", "institution", "integrated_care", "smoking", "drinking", "z_loudness", "tinnitus_location", "tinnitus_pattern", "tinnitus_duration", "hypertension")
)

knots_all <- as.numeric(quantile(dat$social_support, probs = c(0.05, 0.35, 0.65, 0.95), na.rm = TRUE))
inner_knots <- knots_all[c(2, 3)]
boundary_knots <- knots_all[c(1, 4)]
full_covars <- model_terms[["Model 8: exact manuscript fully adjusted"]]
cov_rhs <- paste(full_covars, collapse = " + ")
spline_rhs <- paste0("ns(social_support, knots = c(", paste(inner_knots, collapse = ", "), "), Boundary.knots = c(", paste(boundary_knots, collapse = ", "), "))")

lm_none <- lm(as.formula(paste("thi ~", cov_rhs)), data = dat)
lm_linear <- lm(as.formula(paste("thi ~ social_support +", cov_rhs)), data = dat)
lm_spline <- lm(as.formula(paste("thi ~", spline_rhs, "+", cov_rhs)), data = dat)
glm_none <- glm(as.formula(paste("high_thi ~", cov_rhs)), data = dat, family = binomial())
glm_linear <- glm(as.formula(paste("high_thi ~ social_support +", cov_rhs)), data = dat, family = binomial())
glm_spline <- glm(as.formula(paste("high_thi ~", spline_rhs, "+", cov_rhs)), data = dat, family = binomial())

rcs_tests <- tibble(
  outcome = c("THI score", "THI score", "Severe/catastrophic THI", "Severe/catastrophic THI"),
  test = c("overall association", "nonlinearity", "overall association", "nonlinearity"),
  knots = paste(knots_all, collapse = ", "),
  p = c(
    anova(lm_none, lm_spline)$`Pr(>F)`[2],
    anova(lm_linear, lm_spline)$`Pr(>F)`[2],
    anova(glm_none, glm_spline, test = "Chisq")$`Pr(>Chi)`[2],
    anova(glm_linear, glm_spline, test = "Chisq")$`Pr(>Chi)`[2]
  )
) %>% mutate(p_formatted = fmt_p(p))
write_csv_out(rcs_tests, "12_rcs_tests.csv")

ref_dat <- dat[rep(1, 200), , drop = FALSE]
grid_ss <- seq(min(dat$social_support), max(dat$social_support), length.out = nrow(ref_dat))
ref_dat$social_support <- grid_ss
for (v in c("z_age", "z_bmi", "z_loudness")) ref_dat[[v]] <- 0
for (v in categorical_covars) {
  ref_dat[[v]] <- factor(mode_value(dat[[v]]), levels = levels(dat[[v]]))
}

pred_lm <- predict(lm_spline, newdata = ref_dat, se.fit = TRUE)
plot_lm <- tibble(
  social_support = grid_ss,
  estimate = pred_lm$fit,
  low = pred_lm$fit - 1.96 * pred_lm$se.fit,
  high = pred_lm$fit + 1.96 * pred_lm$se.fit
)
pred_glm <- predict(glm_spline, newdata = ref_dat, type = "link", se.fit = TRUE)
plot_glm <- tibble(
  social_support = grid_ss,
  estimate = plogis(pred_glm$fit),
  low = plogis(pred_glm$fit - 1.96 * pred_glm$se.fit),
  high = plogis(pred_glm$fit + 1.96 * pred_glm$se.fit)
)

plot_glm <- plot_glm %>%
  mutate(across(c(estimate, low, high), ~ .x * 100))

publication_rcs_theme <- function(plot_margin = margin(8, 12, 8, 8)) {
  theme_classic(base_size = 11, base_family = "Arial") +
    theme(
      plot.title = element_text(size = 14, face = "bold", hjust = 0, margin = margin(b = 6)),
      axis.title = element_text(size = 12, color = "black"),
      axis.text = element_text(size = 10.5, color = "black"),
      axis.line = element_line(color = "black", linewidth = 0.35),
      axis.ticks = element_line(color = "black", linewidth = 0.35),
      plot.margin = plot_margin
    )
}

embed_svg_fonts <- function(svg_path) {
  font_regular <- "C:/Windows/Fonts/arial.ttf"
  font_bold <- "C:/Windows/Fonts/arialbd.ttf"
  if (!requireNamespace("base64enc", quietly = TRUE) ||
      !file.exists(font_regular) ||
      !file.exists(font_bold)) {
    return(invisible(FALSE))
  }

  svg_text <- paste(readLines(svg_path, warn = FALSE, encoding = "UTF-8"), collapse = "\n")
  if (grepl("@font-face", svg_text, fixed = TRUE)) {
    return(invisible(TRUE))
  }

  regular_b64 <- base64enc::base64encode(font_regular)
  bold_b64 <- base64enc::base64encode(font_bold)
  font_defs <- paste0(
    "<defs><style type=\"text/css\"><![CDATA[\n",
    "@font-face { font-family: 'Arial'; font-style: normal; font-weight: 400; ",
    "src: url(data:font/truetype;charset=utf-8;base64,", regular_b64, ") format('truetype'); }\n",
    "@font-face { font-family: 'Arial'; font-style: normal; font-weight: 700; ",
    "src: url(data:font/truetype;charset=utf-8;base64,", bold_b64, ") format('truetype'); }\n",
    "]]></style></defs>"
  )

  svg_open <- regexpr("<svg[^>]*>", svg_text)
  if (svg_open[1] == -1) {
    return(invisible(FALSE))
  }
  insert_after <- svg_open[1] + attr(svg_open, "match.length") - 1
  svg_text <- paste0(
    substr(svg_text, 1, insert_after),
    "\n",
    font_defs,
    "\n",
    substr(svg_text, insert_after + 1, nchar(svg_text))
  )
  writeLines(svg_text, svg_path, useBytes = TRUE)
  invisible(TRUE)
}

p1 <- ggplot(plot_lm, aes(social_support, estimate)) +
  geom_ribbon(aes(ymin = low, ymax = high), fill = "#b9d7ee", alpha = 0.65, linewidth = 0) +
  geom_line(color = "#084c8d", linewidth = 0.9) +
  geom_rug(
    data = dat, aes(x = social_support), inherit.aes = FALSE,
    sides = "b", color = "#4b5563", alpha = 0.18, linewidth = 0.25
  ) +
  scale_x_continuous(breaks = scales::breaks_pretty(n = 6), expand = expansion(mult = c(0.02, 0.02))) +
  scale_y_continuous(breaks = scales::breaks_pretty(n = 5)) +
  labs(
    x = "Social support score",
    y = "Adjusted THI score",
    title = "A. THI score"
  ) +
  publication_rcs_theme(margin(8, 24, 8, 8))

p2 <- ggplot(plot_glm, aes(social_support, estimate)) +
  geom_ribbon(aes(ymin = low, ymax = high), fill = "#f6c08a", alpha = 0.65, linewidth = 0) +
  geom_line(color = "#b24b00", linewidth = 0.9) +
  geom_rug(
    data = dat, aes(x = social_support), inherit.aes = FALSE,
    sides = "b", color = "#4b5563", alpha = 0.18, linewidth = 0.25
  ) +
  scale_x_continuous(breaks = scales::breaks_pretty(n = 6), expand = expansion(mult = c(0.02, 0.02))) +
  scale_y_continuous(
    limits = c(0, 104),
    breaks = seq(0, 100, 20),
    labels = scales::label_number(accuracy = 1),
    expand = expansion(mult = c(0, 0))
  ) +
  labs(
    x = "Social support score",
    y = "Predicted probability, %",
    title = "B. Severe/catastrophic THI"
  ) +
  publication_rcs_theme(margin(14, 8, 8, 24))

figure1_rcs <- (p1 + p2) +
  plot_layout(ncol = 2)

figure_width <- 8.0
figure_height <- 4.0

ggsave(file.path(out_dir, "RCS_THI_score.png"), p1, width = 4.2, height = 3.6, units = "in", dpi = 600, bg = "white")
ggsave(file.path(out_dir, "RCS_severe_catastrophic_probability.png"), p2, width = 4.2, height = 3.6, units = "in", dpi = 600, bg = "white")
ggsave(file.path(out_dir, "RCS_social_support_THI_two_panel.png"), figure1_rcs, width = figure_width, height = figure_height, units = "in", dpi = 600, bg = "white")
ggsave(file.path(out_dir, "Figure1_RCS_social_support_THI_600dpi.png"), figure1_rcs, width = figure_width, height = figure_height, units = "in", dpi = 600, bg = "white")
ggsave(file.path(out_dir, "Figure1_RCS_social_support_THI_600dpi.tiff"), figure1_rcs, width = figure_width, height = figure_height, units = "in", dpi = 600, bg = "white", device = "tiff", compression = "lzw")
ggsave(file.path(out_dir, "Figure1_RCS_social_support_THI.pdf"), figure1_rcs, width = figure_width, height = figure_height, units = "in", bg = "white", device = grDevices::cairo_pdf, family = "Arial", fallback_resolution = 600)
svg_path <- file.path(out_dir, "Figure1_RCS_social_support_THI.svg")
ggsave(svg_path, figure1_rcs, width = figure_width, height = figure_height, units = "in", bg = "white", device = svglite::svglite, system_fonts = list(sans = "Arial"))
embed_svg_fonts(svg_path)

figure_legend <- paste0(
  "Figure 1. Restricted cubic spline analyses of the associations between social support and tinnitus-related disability. ",
  "Panel A shows the fully adjusted association between social support score and THI score. ",
  "Panel B shows the fully adjusted association between social support score and the predicted probability of severe/catastrophic THI. ",
  "Solid lines represent adjusted estimates, and shaded areas indicate 95% confidence intervals. ",
  "Rug plots along the x-axis show the distribution of social support scores. ",
  "Models were adjusted for demographic, socioeconomic, lifestyle, institutional, and tinnitus-related clinical covariates. ",
  "Knots were placed at social support scores of 15, 21, 27, and 37. ",
  "P for nonlinearity was <0.001 for both panels."
)
writeLines(figure_legend, file.path(out_dir, "figure_legend_RCS.txt"), useBytes = TRUE)

message("Exported Figure 1 files and figure_legend_RCS.txt")
```
