# Supplementary material for: Higher social support is associated with lower tinnitus-related disability among institutionalized older adults: a cross-sectional study
Source: Front Public Health. 2026 Sep 9;14:1886767. doi: 10.3389/fpubh.2026.1886767 (PMC13597730; doi:10.3389/fpubh.2026.1886767)
Supplement: Supplementary file 2 [file Supplementary_file_2.docx]

**FIG2 final complete R code**

Complete final redraw script for the subgroup forest plot. Generated: 2026-05-11 08:40

```
options(stringsAsFactors = FALSE)

local_lib <- file.path(getwd(), "R_lib")
if (dir.exists(local_lib)) {
  .libPaths(c(local_lib, .libPaths()))
}

shared_lib <- file.path("D:", "耳鸣", "R_local_lib")
if (dir.exists(shared_lib)) {
  .libPaths(c(shared_lib, .libPaths()))
}

suppressPackageStartupMessages({
  library(ggplot2)
  library(tibble)
  library(dplyr)
  library(grid)
})

ge <- intToUtf8(8805)
beta_symbol <- intToUtf8(946)

subgroup_data <- tribble(
  ~group, ~level, ~n, ~beta, ~lower, ~upper, ~p, ~p_interaction,
  "Age", "<80", 185, -5.86, -9.18, -2.55, "<0.001", "0.748",
  "Age", paste0(ge, "80"), 205, -4.67, -7.35, -1.98, "<0.001", "",

  "Sex", "Male", 153, -5.12, -8.77, -1.46, "0.007", "0.648",
  "Sex", "Female", 237, -5.29, -7.93, -2.65, "<0.001", "",

  "Family visit frequency", "At least monthly", 294, -4.23, -6.61, -1.84, "<0.001", "0.156",
  "Family visit frequency", "Quarterly or less", 96, -7.49, -11.69, -3.28, "<0.001", "",

  "Tinnitus duration", "<5 years", 192, -7.86, -10.65, -5.07, "<0.001", "<0.001",
  "Tinnitus duration", paste0(ge, "5 years"), 198, -0.69, -3.20, 1.82, "0.588", "",

  "Tinnitus pattern", "Continuous", 138, -7.71, -11.60, -3.82, "<0.001", "0.123",
  "Tinnitus pattern", "Intermittent", 252, -4.42, -6.89, -1.94, "<0.001", "",

  "Institution ownership", "Public", 234, -3.33, -5.92, -0.74, "0.013", "0.018",
  "Institution ownership", "Private", 156, -8.37, -11.87, -4.87, "<0.001", "",

  "Integrated medical-nursing care", "Yes", 184, -3.56, -6.51, -0.60, "0.019", "0.057",
  "Integrated medical-nursing care", "No", 206, -7.11, -10.00, -4.21, "<0.001", "",

  "Hypertension", "No", 288, -3.78, -6.08, -1.49, "0.001", "<0.001",
  "Hypertension", "Yes", 102, -6.83, -11.26, -2.40, "0.003", "",

  "Tinnitus loudness", "<median", 138, -3.32, -6.58, -0.06, "0.048", "0.267",
  "Tinnitus loudness", paste0(ge, "median"), 252, -6.56, -9.30, -3.82, "<0.001", ""
)

plot_data <- subgroup_data %>%
  mutate(
    row = row_number(),
    y = rev(row),
    group_label = if_else(!duplicated(group), group, ""),
    estimate_ci = sprintf("%.2f (%.2f to %.2f)", beta, lower, upper)
  )

n_rows <- nrow(plot_data)

# The whole figure is drawn in one ggplot coordinate system. This keeps the
# table and forest panel aligned in vector and raster exports.
x_group <- 0.025
x_level <- 0.275
x_n <- 0.405
x_forest_min <- 0.475
x_forest_max <- 0.690
x_estimate <- 0.765
x_p <- 0.875
x_pint <- 0.965

forest_min <- -12
forest_max <- 3
map_forest_x <- function(x) {
  x_forest_min + (x - forest_min) / (forest_max - forest_min) * (x_forest_max - x_forest_min)
}

plot_data <- plot_data %>%
  mutate(
    x_beta = map_forest_x(beta),
    x_lower = map_forest_x(lower),
    x_upper = map_forest_x(upper)
  )

tick_values <- c(-12, -8, -4, 0, 3)
tick_data <- tibble(
  tick = tick_values,
  x = map_forest_x(tick_values),
  y0 = 0.45,
  y1 = 0.68,
  label_y = 0.18
)

row_shading <- tibble(
  y = plot_data$y,
  ymin = plot_data$y - 0.5,
  ymax = plot_data$y + 0.5,
  fill = plot_data$row %% 2 == 0
)

header_y <- n_rows + 1.25
axis_y <- 0.60
axis_label_y <- -0.25

base_family <- "Arial"
text_color <- "#1F2933"
muted_color <- "#52616B"
line_color <- "#D7DEE5"
forest_color <- "#1F6F8B"

final_plot <- ggplot() +
  geom_rect(
    data = filter(row_shading, fill),
    aes(xmin = 0, xmax = 1, ymin = ymin, ymax = ymax),
    fill = "#F7F9FB",
    color = NA
  ) +
  geom_segment(
    data = tibble(y = plot_data$y - 0.5),
    aes(x = 0.015, xend = 0.985, y = y, yend = y),
    linewidth = 0.22,
    color = line_color
  ) +
  geom_segment(
    aes(x = 0.015, xend = 0.985, y = n_rows + 0.55, yend = n_rows + 0.55),
    linewidth = 0.55,
    color = "#9AA6B2"
  ) +
  geom_segment(
    aes(x = map_forest_x(0), xend = map_forest_x(0), y = 0.5, yend = n_rows + 0.5),
    linewidth = 0.35,
    linetype = "dashed",
    color = "#8795A1"
  ) +
  geom_segment(
    aes(x = x_forest_min, xend = x_forest_max, y = axis_y, yend = axis_y),
    linewidth = 0.32,
    color = "#697985"
  ) +
  geom_segment(
    data = tick_data,
    aes(x = x, xend = x, y = y0, yend = y1),
    linewidth = 0.28,
    color = "#697985"
  ) +
  geom_text(
    data = tick_data,
    aes(x = x, y = label_y, label = tick),
    family = base_family,
    size = 2.05,
    color = muted_color
  ) +
  annotate(
    "text",
    x = (x_forest_min + x_forest_max) / 2,
    y = axis_label_y,
    label = paste0(beta_symbol, " per 1-SD increase in social support"),
    family = base_family,
    size = 2.25,
    color = muted_color
  ) +
  geom_segment(
    data = plot_data,
    aes(x = x_lower, xend = x_upper, y = y, yend = y),
    linewidth = 0.72,
    lineend = "round",
    color = forest_color
  ) +
  geom_point(
    data = plot_data,
    aes(x = x_beta, y = y),
    shape = 15,
    size = 2.25,
    color = forest_color
  ) +
  geom_text(
    aes(x = x_group, y = header_y, label = "Subgroup"),
    hjust = 0,
    fontface = "bold",
    family = base_family,
    size = 2.55,
    color = text_color
  ) +
  geom_text(
    aes(x = x_level, y = header_y, label = "Level"),
    hjust = 0,
    fontface = "bold",
    family = base_family,
    size = 2.55,
    color = text_color
  ) +
  geom_text(
    aes(x = x_n, y = header_y, label = "n"),
    hjust = 0.5,
    fontface = "bold",
    family = base_family,
    size = 2.55,
    color = text_color
  ) +
  geom_text(
    aes(x = (x_forest_min + x_forest_max) / 2, y = header_y, label = paste0("Adjusted ", beta_symbol)),
    hjust = 0.5,
    fontface = "bold",
    family = base_family,
    size = 2.55,
    color = text_color
  ) +
  geom_text(
    aes(x = x_estimate, y = header_y, label = paste0(beta_symbol, " (95% CI)")),
    hjust = 0.5,
    fontface = "bold",
    family = base_family,
    size = 2.55,
    color = text_color
  ) +
  geom_text(
    aes(x = x_p, y = header_y, label = "P value"),
    hjust = 0.5,
    fontface = "bold",
    family = base_family,
    size = 2.55,
    color = text_color
  ) +
  geom_text(
    aes(x = x_pint, y = header_y, label = "P interaction"),
    hjust = 0.5,
    fontface = "bold",
    family = base_family,
    size = 2.55,
    color = text_color
  ) +
  geom_text(
    data = plot_data,
    aes(x = x_group, y = y, label = group_label, fontface = if_else(group_label == "", "plain", "bold")),
    hjust = 0,
    family = base_family,
    size = 2.42,
    color = text_color
  ) +
  geom_text(
    data = plot_data,
    aes(x = x_level, y = y, label = level),
    hjust = 0,
    family = base_family,
    size = 2.42,
    color = text_color
  ) +
  geom_text(
    data = plot_data,
    aes(x = x_n, y = y, label = n),
    hjust = 0.5,
    family = base_family,
    size = 2.42,
    color = text_color
  ) +
  geom_text(
    data = plot_data,
    aes(x = x_estimate, y = y, label = estimate_ci),
    hjust = 0.5,
    family = base_family,
    size = 2.35,
    color = text_color
  ) +
  geom_text(
    data = plot_data,
    aes(x = x_p, y = y, label = p),
    hjust = 0.5,
    family = base_family,
    size = 2.35,
    color = text_color
  ) +
  geom_text(
    data = plot_data,
    aes(x = x_pint, y = y, label = p_interaction),
    hjust = 0.5,
    family = base_family,
    size = 2.35,
    color = text_color
  ) +
  coord_cartesian(xlim = c(0, 1), ylim = c(-0.75, n_rows + 1.75), clip = "off") +
  scale_x_continuous(expand = c(0, 0)) +
  scale_y_continuous(expand = c(0, 0)) +
  theme_void(base_family = base_family) +
  theme(
    plot.background = element_rect(fill = "white", color = NA),
    plot.margin = margin(7, 7, 7, 7, unit = "mm")
  )

ggsave(
  "Figure2_subgroup_forest.pdf",
  plot = final_plot,
  width = 180,
  height = 135,
  units = "mm",
  device = cairo_pdf
)

svglite::svglite(
  "Figure2_subgroup_forest.svg",
  width = 180 / 25.4,
  height = 135 / 25.4
)
print(final_plot)
dev.off()

ragg::agg_tiff(
  "Figure2_subgroup_forest.tiff",
  width = 180,
  height = 135,
  units = "mm",
  res = 600,
  compression = "lzw",
  background = "white"
)
print(final_plot)
dev.off()

ragg::agg_png(
  "Figure2_subgroup_forest.png",
  width = 180,
  height = 135,
  units = "mm",
  res = 600,
  background = "white"
)
print(final_plot)
dev.off()

message("Exported Figure2_subgroup_forest.pdf/svg/tiff/png")
```
