# Supplementary material for: Higher social support is associated with lower tinnitus-related disability among institutionalized older adults: a cross-sectional study
Source: Front Public Health. 2026 Sep 9;14:1886767. doi: 10.3389/fpubh.2026.1886767 (PMC13597730; doi:10.3389/fpubh.2026.1886767)
Supplement: Supplementary file 3 [file Table_1.DOCX]

**Supplementary Table S1.** Additional participant characteristics and covariates according to severe/catastrophic tinnitus-related disability

| Characteristic | Overall | Non-severe/catastrophic | Severe/catastrophic | *P* value |
| --- | --- | --- | --- | --- |
| BMI, kg/m² | 23.89± 3.71 | 23.85 ± 3.92 | 23.93 ± 3.47 | 0.825 |
| Marital status |  |  |  | 0.364 |
| Unmarried | 26 (6.7%) | 10 (5.0%) | 16 (8.5%) |  |
| Married | 126 (32.3%) | 61(30.3%) | 65 (34.4%) |  |
| Divorced | 61 (15.6%) | 33 (16.4%) | 28 (14.8%) |  |
| Widowed | 177 (45.4%) | 97(48.3%) | 80 (42.3%) |  |
| Monthly income, RMB |  |  |  | 0.542 |
| <2,000 | 48 (12.3%) | 20 (10.0%) | 28 (14.8%) |  |
| 2,000–4,000 | 124 (31.8%) | 66 (32.8%) | 58 (30.7%) |  |
| 4,000–6,000 | 154 (39.5%) | 81 (40.3%) | 73 (38.6%) |  |
| >6,000 | 64 (16.4%) | 34 (16.9%) | 30 (15.9%) |  |
| Medical insurance type |  |  |  | 0.515 |
| Self-pay | 16 (4.1%) | 6 (3.0%) | 10 (5.3%) |  |
| Resident insurance | 91 (23.3%) | 47 (23.4%) | 44 (23.3%) |  |
| Employee insurance | 283 (72.6%) | 148 (73.6%) | 135 (71.4%) |  |
| Tinnitus location |  |  |  | 0.602 |
| Left ear only | 150 (38.5%) | 76 (37.8%) | 74 (39.2%) |  |
| Right ear only | 88 (22.6%) | 41 (20.4%) | 47 (24.9%) |  |
| Bilateral | 76 (19.5%) | 43 (21.4%) | 33 (17.5%) |  |
| Cranial tinnitus | 76 (19.5%) | 41 (20.4%) | 35 (18.5%) |  |
