# Supplementary material for: Higher social support is associated with lower tinnitus-related disability among institutionalized older adults: a cross-sectional study
Source: Front Public Health. 2026 Sep 9;14:1886767. doi: 10.3389/fpubh.2026.1886767 (PMC13597730; doi:10.3389/fpubh.2026.1886767)
Supplement: Supplementary file 4 [file Table_2.docx]

**Supplementary Table S2. Association between perceived social support and tinnitus-related disability after additional adjustment for hearing-related variables**

| Outcome | Social support effect | Self-reported hearing loss | Hearing aid use | Professional tinnitus treatment | Joint test |
| --- | --- | --- | --- | --- | --- |
| THI total score | β＝−5.39 (−7.49～−3.28), P<0.001 | β＝0.35 (−3.62～4.31)，P＝0.864 | β＝4.03 (−0.53～8.59)，P＝0.083 | β＝1.48 (−2.55～5.51)，P＝0.471 | P＝0.208 |
| Moderate-or-worse THI | **OR＝0.59 (0.40～0.87),**  P**＝0.007** | **OR＝0.70 (0.33～1.47), P＝0.348** | **OR＝1.88 (0.79～4.49), P＝0.153** | **OR＝0.62 (0.30～1.28), P＝0.197** | **P＝0.301** |
| Severe/catastrophic THI | **OR＝0.58 (0.42～0.80),**  P<0.001 | **OR＝1.13 (0.58～2.21), P＝0.723** | **OR＝1.70 (0.79～3.65), P＝0.171** | **OR＝1.61 (0.81～3.22), P＝0.176** | **P＝0.159** |

**Note.** β and OR represent the effect per 1-SD higher SSA-13 score or the effect of each hearing-related variable, as applicable. THI total score was the primary outcome, severe/catastrophic THI was an additional binary outcome, and moderate-or-worse THI was examined using an alternative threshold in the sensitivity analysis. All models were based on the fully adjusted covariate set and additionally included self-reported hearing loss, hearing aid use, and receipt of professional treatment for tinnitus. HC3 robust standard errors were used. Hearing aid use was dichotomized as any use versus none, and professional treatment was categorized as any reported professional tinnitus treatment versus none. Self-reported hearing loss was not confirmed by audiometric testing.
