# Supplementary material for: Higher social support is associated with lower tinnitus-related disability among institutionalized older adults: a cross-sectional study
Source: Front Public Health. 2026 Sep 9;14:1886767. doi: 10.3389/fpubh.2026.1886767 (PMC13597730; doi:10.3389/fpubh.2026.1886767)
Supplement: Supplementary file 5 [file Table_3.DOCX]

**Supplementary Table S3.** Associations between perceived social support and THI subscale scores

| **THI**  **Dimension** | **Items (range)** | **Mean ± SD** | **Adjusted β** | **95% CI** | **Standardized β** | ***P*** |
| --- | --- | --- | --- | --- | --- | --- |
| Functional | 11 (0–44) | 24.23±9.98 | −2.15 | −3.18 to −1.12 | −0.216 | <0.001 |
| Emotional | 9 (0–36) | 20.06±8.47 | −1.98 | −2.87 to −1.10 | −0.234 | <0.001 |
| Catastrophic | 5 (0–20) | 10.85±4.78 | −1.10 | −1.56 to −0.64 | −0.230 | <0.001 |

Notes: β represents the unstandardized change in subscale score per 1-SD higher SSA-13 score. Models were adjusted for age, sex, education level, body mass index, marital status, income, medical insurance type, smoking history, drinking history, family visit frequency, tinnitus duration, tinnitus pattern, tinnitus location, tinnitus loudness, facility ownership, availability of integrated medical and elderly care services, and hypertension. HC3 robust standard errors were used. Standardized β values are provided to facilitate comparison across subscales with different score ranges.
